# Hospital Meetings, &C.

**Published:** 1898-06-25

**Authors:** 


### HOSPITAL MEETINGS, &C.

### THE PRINCE OF WALES AT UNIVERSITY COLLEGE HOSPITAL.

H.R.H. Lays the Foundation Stone.

H.R.H. the Prince of Wales, who was accompanied by the Princess of Wales, visited the University College Hospital on Tuesday afternoon in order to lay the foundation stone of the new building, which, through the generosity of Sir J. Blundell Maple, Bart., M.P., is being erected at a cost of some £100,000.

A brilliant company assembled to witness the ceremony, the guests who had accepted invitations including: The Duchess of Bedford, Lady Maple, Lady Reay, Sir Savile and Lady Crossley, Sir H. C. Burdett, the Chief Rabbi, Dr. Collins, L.C.C., Professor Victor Horsley, Lord Lister, P.R.S., Lady Jessel, and many other well-known people. On arriving at the temporary platform His Royal Highness was thanked by the Duke of Bedford, president of the hospital, for attending to lay the stone, and Mr. Henry Lucas, chairman of the Hospital Committee made a short statement in reference to the rebuilding, in the course of which he remarked that at a time when the funds were not sufficient to allow of more than a very small portion of the new hospital being built at first, the committee were agreeably startled by the proposal of Sir Blundell Maple to erect the entire building at his own expense. "Its completion," Mr. Lucas added, "must be gradual on account of the necessity of making provision for the sick poor of the district, and the consequent difficulty in pulling down the present building until a portion of the new hospital is ready for occupation. We trust, however, that the whole of the new building will be completed within three or four years, and we feel confident that it will then be of lasting benefit to the poor and the means of development of the great school of medicine associated with University College."

At the conclusion of Mr. Lucas's statement His Royal Highness said: My Lord Duke, Ladies and Gentlemen,—The Princess of Wales and I feel much pleasure in coming here to-day in order that I may lay the foundation-stone of the building which Sir Blundell Maple has so generously undertaken to erect for the University College Hospital. I learn that he was prompted to make this munificent offer, involving the expenditure of upwards of £100,000, by his sympathy with the poor of a district with which he has long been associated, and by his earnest desire to enable them to obtain medical and surgical assistance at times when it is most needed, but when they are least able, if unaided, to defray its expense. No such magnificent gift for the sick poor of London has, I believe, been made since Thomas Guy in the seventeenth century established the hospital which bears his name. The needy of the locality should indeed be thankful that Sir Blundell Maple, who has shown himself so anxious to promote their welfare, is living in their midst. The prosperous will I trust practically mark their appreciation of his generosity by their liberal support of the hospital he is building and by contributing to alleviate the sufferings of the sick and indigent. I ask subscribers to remember that all general collections for hospitals ought to be regarded as supplements to and not as substitutes for local subscriptions. Of the utility and the important requirements which the University College meets there can be no doubt. In its immediate vicinity there exists a large population of the labouring class, many of whom have difficulty even when they are in full work in maintaining themselves and their families. When incapacitated by illness or an accident it is impossible for them to provide themselves with the means of medical relief. I would also point out how valuable are the resources of the hospital when grave accidents occur in connection with the three large railway companies, the London and North-Western, the Midland, and the Great Northern, which all have their London stations within a short distance of the place where the sufferers can obtain immediate relief. Let me say a few words on the incalculable value of the hospital as a school of medicine. Several thousands of its graduates are now putting into practice in various parts of the world the lessons they learned at this institution. Many students have risen to great distinction in their profession, and foremost among these I am proud to reckon my old friend, Sir William Jenner, who is universally recognised as one of the most eminent medical men of the nineteenth century. I am sure you will unreservedly agree with me that the new building will increase in an immense degree the benefits derived from this hospital. The additional accommodation for the patients and the material improvements in the medical, surgical, and nursing arrangements, as well as in various details of administration, must commend themselves to the approval of all who have studied the question. In all the advantages which I have enumerated the medical school will fully participate. When the new building is completed the training of pupils will be carried on under far more favourable circumstances than hitherto. By continuing to study the treatment of patients according to the newest improvements, regulated by the most modern science, the students cannot fail to acquire a practical knowledge advantageous to themselves and ultimately of the highest benefit to the general public. (Cheers.)

Sir Blundell Maple came forward at the conclusion of the Prince's speech and expressed to His Royal Highness how dear the desire was to his (Sir Blundell's) heart that the hospital should be a great boon to the suffering and also a school where medical men should be trained for their life's work. He further referred to the great and practical interest the Prince had shown in London hospitals, and hoped that His Royal Highness would continue to exercise his widespread influence to raise further money for the aid of those who may be in sickness and trouble.

Amid loud applause the Prince then stepped forward and laid the foundation stone of the edifice, and after the Bishop of London had offered up a prayer, the Royal party left, followed by the cheers of the assembly. The stone bore the following inscription: "This stone was laid by His Royal Highness Albert Edward Prince of Wales, K.G., vice-patron of the University College Hospital, on Tuesday, June 21st, 1898, as a memorial of the rebuilding of this hospital through the generosity of Sir J. Blundell Maple, Bart., M.P."

### NORTH-EASTERN HOSPITAL FOR CHILDREN.

Lord Amherst of Hackney presided on Friday at a public meeting at Hackney Town Hall called to demonstrate the urgent need that exists for an increase of accommodation at the North-Eastern Hospital for Children. Sir Andrew Scoble, M.P., proposed, and Mr. W. R. Bousfield, M.P., seconded, a resolution affirming that it was necessary in the public interest that the completion of this hospital's premises which had been left unfinished since 1880 should no longer be delayed. The Rev. Prebendary Shelford (rector of Stoke Newington and chairman of Stoke Newington Vestry), in supporting the resolution, stated that the districts contiguous to the hospital would provide the funds for maintaining the new wards if the wealthy districts of London would build them. Many other speakers representative of Hackney followed, and it appeared that the hospital at present contained 57 beds, which it was intended to increase to over 100 at a cost of about £20,000, £8,500 of which had already been obtained. Increased accommodation was required in every department of the hospital in order to more efficiently meet the growing demands of the rapidly-increasing population of the surrounding districts, which already contained nearly half a million inhabitants, mostly of the industrial classes, who were almost entirely dependent on this hospital for the treatment of their children in sickness and accident.

### CENTRAL LONDON OPHTHALMIC HOSPITAL.

The Lord Mayor was to have presided at the meeting in aid of the Central London Ophthalmic Hospital at the Mansion House on Thursday, 16th inst., but, in his unavoidable absence, Alderman Sir Joseph Savory, M.P., took the chair, the Lady Mayoress, however, being present.

In his opening remarks, the Chairman said that though not a crowded meeting, it was, he felt sure, an enthusiastic one. The hospital to assist which they were gathered had been in existence since 1843, and during that period had benefited and relieved some 400,000 individuals, the number of patients being now about 11,000 yearly. The work, which was done among a very poor population chiefly engaged in the clock and watch trade, in which, of course, the eyesight was of the utmost importance, was well worthy of liberal support. He called upon Lord Brougham and Vaux to propose the first resolution, which was to the effect that the meeting desired to record its sense of the importance and usefulness of the institution, both in regard to the relief afforded and as a school for post-graduate teaching. His Lordship urged the inadequacy of the present building, and said he was glad to know the new lease had been agreed upon. He was quite sure that though, comparatively small, the hospital, under its able management, had more chance of doing good in its special sphere than the larger general hospitals.

Colonel Woodroffe Boyce, who seconded the resolution, put the economic case for support in a nutshell. The people, he said, were either ratepayers or rate supported. If the eye troubles of those engaged in industry were not attended to, they ceased to be the former and became the latter, and so added to the burden of ratepayers generally. In addition to this, he pointed out that many who never entered the hospital benefited by the knowledge and experience gained by medical men there.

Sir Dyce Duckworth, the consulting physician to the hospital, moved a resolution approving the scheme for re-building, and pledging the meeting to do all in its power to further it and so help to alleviate much suffering. He reminded his hearers that the present building was old and inadequate, and must come down. The new project would enable them to enlarge and adapt it to the requirements of to-day in regard to light, air, sanitary arrangements, and also the operating theatre. In that home of charity they were called to make known the needs of an institution which did a very large amount of good and no possible harm. From personal experience he could assure them there was no fear of pauperising people there. It was his belief that the general charges in regard to abuses by out-patients of benefits intended for a more needy class were enormously exaggerated, and were, in the case of well-conducted hospitals, practically nil, but in this case they did not apply at all. Nor did the work of the hospital interfere with the ordinary general practitioner, who was only too glad to promptly consign to it all cases of eye trouble that came in his way. He might say that ophthalmic trouble was one of the few "specialities" the medical profession did recognise, though there were many they did not—a fact the public did not seem to be aware of. When the public wished to pay a compliment to a doctor they called him a specialist, but that term to him (Sir Dyce) was almost degrading and insulting. But in the case of ophthalmic trouble the profession did recognise specialism in the sense that it required special gifts and experience, but these must be grafted on to a thorough general education and training. And there again the ophthalmic hospital was of benefit, since it afforded opportunities to senior or post-graduate students, not incommoded by crowds of other students, as must be the case in the large general hospitals. That the finances of the Ophthalmic were administered with strict economy and perfect propriety was assured by the fact that they received grants from the Hospital Sunday Fund.

Colonel Tatham having seconded the resolution, the Secretary (Mr. Bryant) read a list of donations received at the meeting or previously amounting to £637. The sum of £2,000 has already been received towards the building fund out of an estimated requirement of £15,000.

The meeting closed with a vote of thanks to the Lord Mayor for the use of the Mansion House, in moving which Mr. Brittin Archer, the senior surgeon of the hospital, said they had been told by some people that in advocating this movement they were flogging a dead horse. They were not, however, so dead as these people wished, "and," said he, "we are more likely to be jubilant mourners at their funeral than they at ours," whereat the meeting laughed and dispersed.

